# Flow adjusted transmitral pressure gradient as a modified indicator of functional mitral stenosis after repair for degenerative mitral regurgitation

**DOI:** 10.1111/jocs.16373

**Published:** 2022-03-02

**Authors:** Arudo Hiraoka, Akihiro Hayashida, Toshinori Totusgawa, Misako Toki, Genta Chikazawa, Hidenori Yoshitaka, Taichi Sakaguchi

**Affiliations:** ^1^ Department of Cardiovascular Surgery The Sakakibara Heart Institute of Okayama Okayama Japan; ^2^ Department of Cardiology The Sakakibara Heart Institute of Okayama Okayama Japan; ^3^ Department of Clinical Laboratory The Sakakibara Heart Institute of Okayama Okayama Japan

**Keywords:** diagnostic imaging, echocardiography, mitral regurgitation, mitral stenosis

## Abstract

**Background and Aim:**

After repair of degenerative mitral regurgitation (DMR), the focus is on functional mitral stenosis (FMS) when there is a decline of mitral hemodynamics. Yet, the clinical impacts and a standardized definition are still undecided. Since common mitral hemodynamic parameters are influenced by transmitral flow, the aim of this study is to seek the impact of flow adjusted transmitral pressure gradient (TMPG) by left ventricular stroke volume (LVSV) on the midterm outcomes.

**Methods:**

Three hundred one patients who had undergone isolated mitral valve repair for degenerative lesions with annuloplasty prosthesis between October 2012 and June 2019 were included. Postoperative adverse events occurred in 20 patients (6.6%). Flow adjusted TMPG was defined as TMPG/LVSV.

**Results:**

Common mitral hemodynamic parameters were not associated with adverse events. By multivariable analysis, patients’ age, left ventricular ejection fraction (LVEF) and mean TMPG/LVSV were isolated as independent predictors (adjusted hazard ratio: 1.05, 0.95, and 1.16; *p* = .037, .005, and .035). Flow adjusted TMPG was significantly higher in the full ring group compared to the partial band group (0.051 mmHg/ml, [0.038–0.068] vs. 0.041 mmHg/ml, [0.031–0.056]; *p* < .001) and had a significantly negative correlation with the size of the annuloplasty prosthesis (*r* = −0.37, *p* < .001).

**Conclusions:**

Conventional mitral hemodynamic parameters were not associated with adverse cardiac events after repair for DMR. Adjustment by flow has a potential to advance pressure gradient to a more sensitive indicator of FMS associated with clinical outcomes.

## 1 INTRODUCTION

Mitral valve repair with annuloplasty prosthesis is a preferred technique for repair of functional and degenerative mitral regurgitation (DMR). Long‐term success of surgical repair results from prevention of recurrent mitral regurgitation (MR). This has been attempted by focusing on restrictive annuloplasty for ischemic functional MR by reducing the mitral effective valve orifice area (EOA). At the same time, this may cause functional mitral stenosis (FMS) and elevation of the transmitral pressure gradient (TMPG). Mitral hemodynamic decline (EOA < 1.5 cm^2^ or mean TMPG > 5 mmHg) has recently been reported in patients with FMS.^1^, ^2^, ^3^, ^4^ Regarding DMR, the remodeling of the mitral annulus with a full ring and the resection of the posterior mitral leaflet sometimes reduced the valve opening area and restricted the excursion of the leaflet.^5^, ^6^ Chan et al.^6^ reported a slight elevation of mean TMPG (>3 mmHg) which can lead to worsening intracardiac hemodynamics, as was observed in 98% of patients (41/42) with full ring.

Additionally, TMPG after mitral valve repair for DMR is reported to cause lower exercise capacity and atrial fibrillation.^6^, ^7^, ^8^ However, several studies have failed to demonstrate an effect of TMPG on adverse events even after undersized annuloplasty for ischemic MR in patients with lower cardiac output.^9^, ^10^, ^11^

On the other hand, TMPG, one of the most common indicators of FMS, is a conflicting echocardiographic parameter. An increase of transmitral flow provided by the left ventricular and atrial output elevates TMPG, resulting in paradoxical FMS.^9^, ^12^ Since greater left ventricular stroke volume (LVSV) increases TMPG, a correlation between flow and gradient should be considered when evaluating the clinical impacts of FMS. We have recently reported that conventional mitral hemodynamic parameters were not associated with adverse cardiac events after annuloplasty for ischemic functional MR. However, flow adjusted TMPG was identified as an independent predictor, and risk stratification by peak TMPG and LVSV predicted midterm outcomes well.^13^ Flow adjusted TMPG may be applied as a predictor of clinical outcomes, even though the left ventricular function is usually preserved in patients with DMR.

Based on these backgrounds, we hypothesized that TMPG adjusted by LVSV can be a more sensitive indicator of FMS associated with adverse events after mitral repair for DMR. The aim of this study is to seek mitral hemodynamic indices (including flow adjusted TMPG) relevant to the midterm outcomes after repair for DMR.

## 2 MATERIALS AND METHODS

### 2.1 Patient population

This study is a retrospective evaluation of mitral valve hemodynamic status measured by resting echocardiogram at several weeks after surgery. Three hundred six patients underwent isolated mitral valve repair for degenerative lesions with annuloplasty prosthesis at the Sakakibara Heart Institute of Okayama between October 2012 and June 2019. After excluding 5 patients with residual mitral regurgitation ≥ moderate, the remaining 301 patients were enrolled. Primary endpoints were defined as postoperative adverse events during follow‐up (stroke, readmission due to de novo arrhythmia, readmission due to heart failure, and reoperation for FMS). This study was approved by the institutional ethics committee in accordance with the ethical standards laid down in the 1964 Declaration of Helsinki and its later amendments (No. A201908‐01, September 26, 2019). Consent for using patients’ data was obtained from all patients. It was not appropriate or possible to involve patients or the public in the design, or conduct, or reporting, or dissemination plans of our research.

### 2.2 Surgical technique of mitral valve repair

Surgical characteristics are presented in Table 2. The main technique of mitral repair included resection in 115 patients (38%), artificial chorda in 165 patients (55%), folding plasty in 18 patients (6%), and augmentation in 3 patients (1%). The prolapse lesion included 49 anterior (16%), 213 posterior (71%) and 39 bi‐leaflet (13%). In this cohort, 76.1% of patients underwent minimally invasive mitral repair via a right mini‐thoracotomy.

### 2.3 Quantitative echocardiography

Standard transthoracic echocardiography was performed on all patients in the left lateral decubitus position by experienced sonographers before and after symptom‐limited exercise, using a commercially available ultrasound machine (Aplio Artida, Toshiba Medical Systems Corporation). Standard data, such as left ventricular ejection fraction (LVEF), end‐diastolic and systolic dimensions, and tricuspid regurgitation pressure gradient (TRPG) were obtained from the official echocardiographic report.^13^ Stroke volume was measured from the LV outflow tract (LVOT) area × LVOT velocity time integral (VTI) by the pulse wave Doppler method. To evaluate the hemodynamic status of the mitral valve, EOA calculated with the continuity equation, peak and mean TMPG, Doppler velocity index (DVI = VTI_MV_/VTI_LVOT_), and peak flow velocity was measured based on standard guidelines.^14^ Additionally, peak and mean TMPG/LVSV were used to adjust the influence of flow. The systolic pulmonary artery pressure (SPAP) was estimated from the maximal velocity of the tricuspid regurgitant jet using continuous‐wave Doppler with the simplified Bernoulli equation and adding the right atrial pressure, which was estimated by the diameter and collapsibility of the inferior vena cava (IVC). For an IVC with diameter <2.1 cm that collapses ≥50% with a sniff, the RAP value of 3 mmHg was used, an IVC with diameter ≥2.1 cm that collapses <50% suggests RAP of 15 mmHg. If IVC diameter and collapse did not fit this scenario, an intermediate value of 8 mmHg was used.^15^, ^16^, ^17^, ^18^

### 2.4 Statistical analysis

Continuous data are presented as median and interquartile values (the first to the third quartile). Categorical variables are given as a count and percentage of patients. Continuous data were compared with a Mann–Whitney *U*‐test. Categorical variables are given as a count and percentage of patients and were compared using the *χ* ^2^ test. When any expected frequency was less than 1, or 20% of expected frequencies were less than or equal to 5, Fisher's exact test was used. Risk factors for cardiac adverse events were evaluated by multivariable Cox proportional hazard regression after univariable analysis. Freedom from adverse events after surgery was compared by the Kaplan–Meier model and the log‐rank test. All data were analyzed using the Statistical Analysis Systems software JMP 12.0 (SAS Institute Inc.).

## 3 RESULTS

### 3.1 Patient characteristics

Patient characteristics are presented in Table 1. The overall median age of patients was 60 (48–68) years, of which 35% (106/301) were female. The median of body surface area was 1.66 m^2^ (1.50–1.78). A partial band was used for 158 (52.5%) patients and a full ring for 143 (47.5%) patients. For the partial band group, CG future (Medtronic Inc.), Cosgrove (Edwards Lifesciences), Duran (Medtronic Inc.), and Tailor (Abbott Laboratories) bands were implanted in 23, 82, 20, and 33 patients, respectively. For the full ring group, 68 Carpentier‐Edwards Physio/Physio II (Edwards Lifesciences) and 75 Memo 3D (LivaNova) rings were implanted.

**Table 1:** Patient baseline characteristics

Variables	(*n* = 301)
Age (years)	60 (48–68)
Female sex	106 (35%)
Body surface area (m^2^)	1.66 (1.50–1.78)
Paroxysmal AF	52 (17%)
Chronic AF	32 (11%)
Hypertension	122 (41%)
Dyslipidemia	88 (29%)
Diabetes mellitus	32 (11%)
COPD	18 (6%)
Old cerebral infarction	10 (3%)

Abbreviations: AF, atrial fibrillation; COPD, chronic obstructive pulmonary disease.

**Table 2:** Surgical characteristics

Surgical characteristics	(*n* = 301)
Right mini‐thoracotomy approach	230 (76.1%)
*Main technique*	
Resection	115 (38%)
Artificial chorda	165 (55%)
Folding	18 (6%)
Augmentation	3 (1%)
*Main lesion*	
Anterior leaflet	49 (16%)
Posterior leaflet	213 (71%)
Bi‐leaflet	39 (13%)

### 3.2 Postoperative echocardiographic data

Postoperative echocardiographic data was shown in Table 3. The median of postoperative LVEF and LVSV was 59% (54–64) and 58 ml (50–68), respectively. The median of SPAP was 22 mmHg (19–27). Regarding mitral hemodynamic parameters, the median of DVI, EOA, EOAI, peak velocity, peak and mean TMPG was 2.1 (1.8–2.5), 1.73 cm^2^ (1.44–2.09), 1.04 cm^2^/m^2^ (0.89–1.30), 1.28 m/s (1.08–1.53), 6.6 mmHg (4.7–9.3), and 2.6 mmHg (2.0–3.4), respectively.

**Table 3:** Postoperative echocardiographic data

Variables	(*n* = 301)
LVDD (mm)	45 (42–49)
LVSD (mm)	31 (28–35)
LVEF (%)	59 (54–64)
LAD (mm)	36 (31–41)
LVSV (ml)	58 (50–68)
LVSVI (ml/m^2^)	35 (31–41)
DVI	2.1 (1.8–2.5)
EOA (cm^2^)	1.73 (1.44–2.09)
EOAI (cm^2^/m^2^)	1.04 (0.89–1.30)
Peak velocity (m/s)	1.28 (1.08–1.53)
Peak TMPG (mmHg)	6.6 (4.7–9.3)
Mean TMPG (mmHg)	2.6 (2.0–3.4)
TRPG (mmHg)	19 (16–23)
SPAP (mmHg)	22 (19–27)

Abbreviations: DVI, doppler velocity index; EOA, effective orifice area; EOAI, EOA index; LAD, left atrial dimension; LVDD, left ventricular diastolic dimension; LVEF, left ventricular ejection fraction; LVSD, left ventricular systolic dimension; LVSV, left ventricular stroke volume; LVSVI, LVSV index; SPAP, systolic pulmonary artery pressure; TMPG, transmitral pressure gradient; TRPG, tricuspid regurgitation pressure gradient.

### 3.3 Predictive parameters of midterm adverse events after repair of DMR

Postoperative adverse events during follow‐up occurred in 20 patients (6.6%), and the adverse events included: 4 stroke, 4 readmission due to de novo arrhythmia, 6 readmission due to heart failure, and 6 reoperation for FMS. The overall 1‐year and 5‐year freedom from adverse events rate was 96.3% and 84.0%, respectively.

Univariable Cox proportional hazard analysis revealed no significant correlation in common mitral hemodynamic parameters (DVI, EOA, EOA index, peak velocity, peak and mean TMPG). Patients’ age (hazard ratio [HR], 1.05; 95% confidence interval [CI], 1.01–1.10; *p* = .006), LVEF (HR, 0.95; 95% CI, 0.92–0.99; *p* = .016), left atrial dimension (HR, 1.09; 95% CI, 1.03–1.14; *p* = .004), TRPG (HR, 1.11; 95% CI, 1.04–1.17; *p* = .001), SPAP (HR, 1.11; 95% CI, 1.05–1.16; *p* < .001), and mean TMPG/LVSV (HR, 1.19; 95% CI, 1.04–1.35; *p* = .014) were detected as risk factors for adverse events. After multivariable analysis, patients’ age (adjusted HR, 1.05, 95%CI; 1.00‐1.10; *p* = .037), LVEF (adjusted HR, 0.95; 95% CI, 0.92–0.99; *p* = .005) and mean TMPG/LVSV (adjusted HR, 1.16; 95% CI, 1.01–1.32; *p* = .039) were isolated as independent predictors of adverse events after repair for DMR. Univariable and multivariable Cox proportional hazard analysis data are shown in Table 4.

**Table 4:** Univariable and multivariable Cox hazard analysis for risk of adverse events

	Univariable			Multivariable		
	HR	95% CI	*p*	Adjusted HR	95% CI	*p*
Age (per 1 year)	1.05	1.01–1.10	.006	1.05	1.00–1.10	.037
Male	0.97	0.40–2.59	.96			
Chronic atrial fibrillation	2.20	0.63–6.00	.20			
Annuloplasty size (per 1 mm)	0.84	0.69–1.02	.08			
Full ring (vs. partial band)	1.24	0.50–3.10	.63			
LVEF (per 1%)	0.95	0.92–0.99	.016	0.95	0.92–0.99	.005
LAD (per 1 mm)	1.09	1.03–1.14	.004			
LVSV (per 1 ml)	0.98	0.95–1.01	.21			
LVSVI (per 1 ml)	0.97	0.92–1.02	.29			
DVI (per 1)	0.98	0.40–2.21	.96			
EOA (per 1 cm^2^)	0.99	0.40–2.24	.98			
EOAI (per 1 cm^2^/m^2^)	1.07	0.23–4.36	.93			
Peak velocity (per 1 m/s)	2.57	0.76–7.89	.13			
Peak TMPG (per 1 mmHg)	1.08	0.98–1.16	.12			
Mean TMPG (per 1 mmHg)	1.24	0.97–1.52	.08			
TRPG (per 1 mmHg)	1.11	1.04–1.17	.001			
SPAP (per 1 mmHg)	1.11	1.05–1.16	<.001			
Peak TMPG/LVSV (per 0.01)	1.05	0.99–1.08	.06			
Mean TMPG/LVSV (per 0.01)	1.19	1.04–1.35	.014	1.16	1.01–1.32	.039

Abbreviations: CI, confidence interval; DVI, doppler velocity index; EOA, effective orifice area; EOAI, EOA index; HR, hazard ratio; LAD, left atrial dimension; LVEF, left ventricular ejection fraction; LVSV, left ventricular stroke volume; LVSVI, LVSV index; SPAP, systolic pulmonary artery pressure; TMPG, transmitral pressure gradient; TRPG, tricuspid regurgitation pressure gradient.

**Figure 1:**
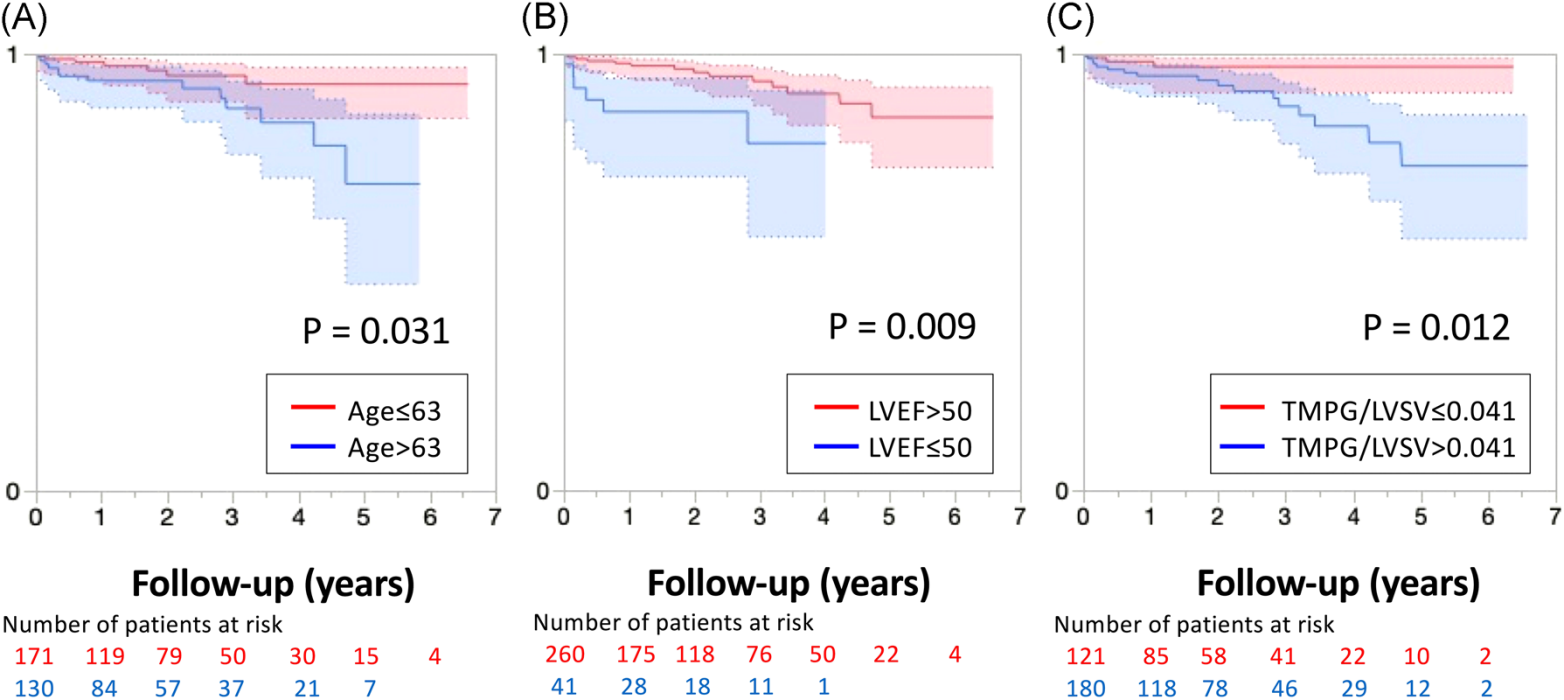
Patients’ age, postoperative left ventricular ejection fraction (LVEF) and mean transmitral pressure gradient adjusted by LV stroke volume (TMPG/LVSV) were detected as independent risks of adverse events after repair of degenerative mitral regurgitation. The Kaplan–Meier curve revealed a significant difference in 5‐year freedom from adverse events rate (A, B, and C)

Patients’ age, postoperative LVEF, and mean TMPG/LVSV were dichotomized at the best cutoff value obtained by receiver operating characteristic analysis as the threshold (63 years, 50%, and 0.041 mmHg/ml). The Kaplan‐Meier curve revealed a significant difference in the 5‐year freedom from adverse events rate (log‐rank test, *p* = .031, .009, and .012; Figure 1A–C).

### 3.4 Stratification of midterm outcomes by mean TMPG and LVEF

**Figure 2:**
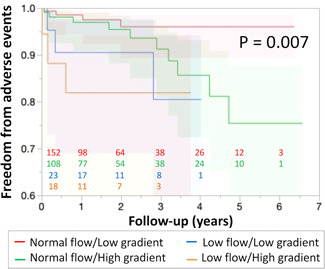
Stratification of midterm outcomes by mean transmitral pressure gradient and left ventricular ejection fraction. The Kaplan–Meier curve revealed a significant difference in the 5‐year freedom from adverse events rate among patients with respective grades of gradient and flow

Based on the best cutoff score discriminating for midterm adverse events, LVEF ≤ 50% and mean TMPG ≥ 3 mmHg were used for stratification to four groups (normal group: LVEF > 50% and mean TMPG < 3 mmHg; normal flow high gradient group: LVEF > 50% and mean TMPG ≥ 3 mmHg; low flow normal gradient group: LVEF ≤ 50% and mean TMPG < 3 mmHg; and, low flow high gradient group: LVEF ≤ 50% and mean TMPG ≥ 3 mmHg). The Kaplan–Meier curve revealed a significant difference in 5‐year freedom from adverse events rate (Wilcoxon test, *p* = .007; Figure 2).

### 3.5 Procedural risk of elevation of flow adjusted TMPG

**Figure 3:**
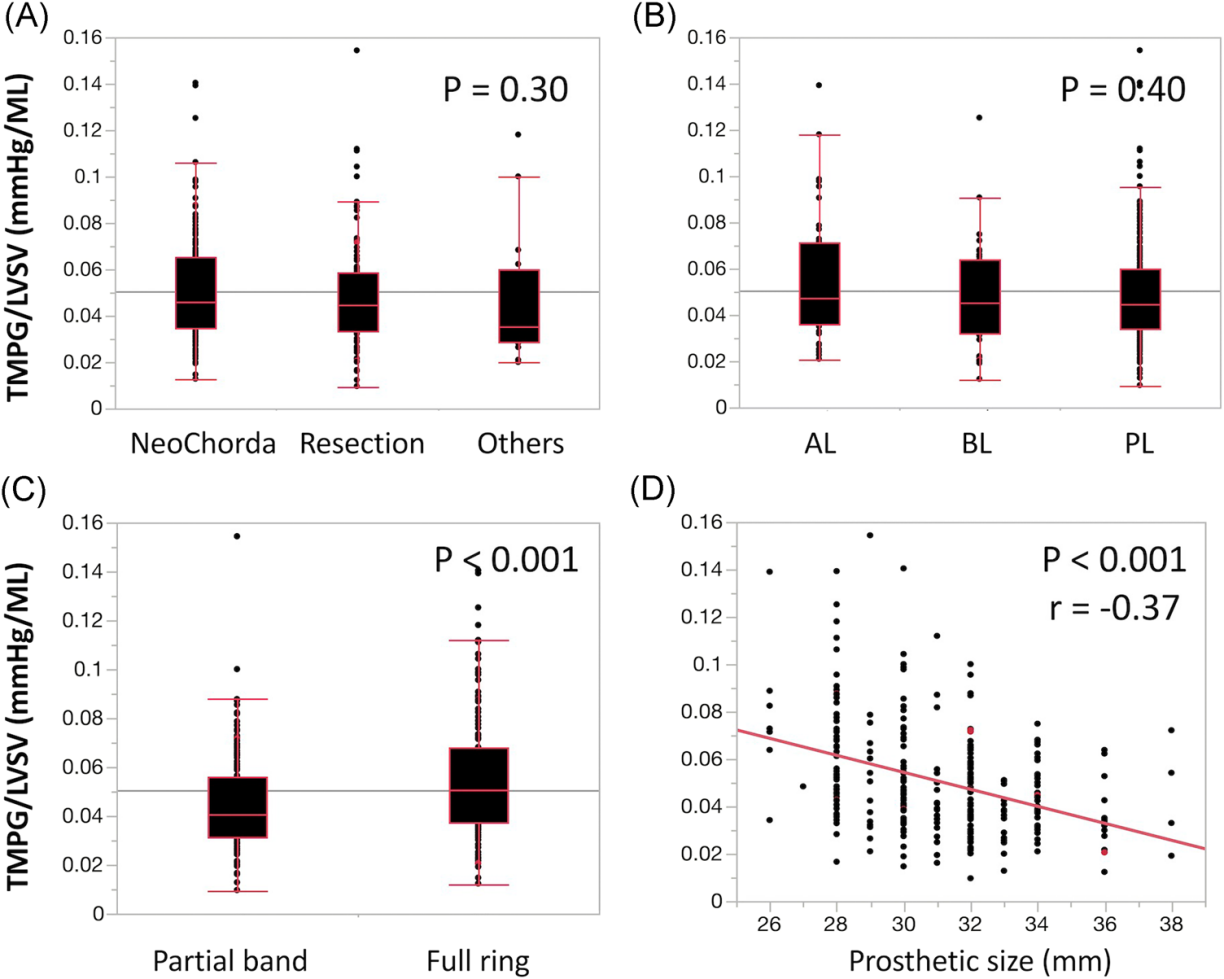
The correlation between mean transmitral pressure gradient adjusted by LV stroke volume (TMPG/LVSV) and procedural factors ([A] repair techniques, [B] the location of lesions [anterior leaflet, AL; bileaflet, BL; posterior leaflet, PL], [C] partial band vs. full ring and [D] size of annuloplasty prosthesis)

**Figure 4:**
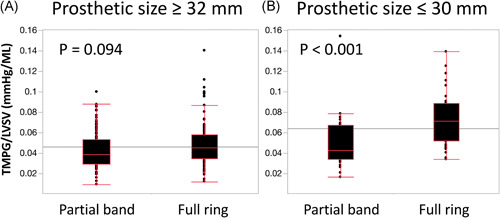
Mean transmitral pressure gradient adjusted by left ventricular stroke volume (TMPG/LVSV) was equivalent in patients with larger annuloplasty prostheses (≥32 mm) (A), but significantly greater in patients with smaller full ring (≤30 mm) (B)

Figure 3 shows the correlation between flow adjusted TMPG and procedural factors. There was no significant difference in flow adjusted TMPG among respective repair techniques (resection, artificial chorda, and others; *p* = .30). The location of lesions was not correlated with flow adjusted TMPG (*p* = .40). Flow adjusted TMPG was significantly higher in the full ring group compared to the partial band group (0.051 mmHg/ml, [0.038–0.068] vs. 0.041 mmHg/ml, [0.031–0.056]; *p* < .001) and had a significantly negative correlation with the size of annuloplasty prosthesis (Pearson's correlation coefficient, *r* = −0.37; *p* < .001). Flow adjusted TMPG was significantly greater in patients with a smaller full ring (≤30 mm), but equivalent in patients with larger annuloplasty prostheses (≥32 mm; Figure 4).

## 4 DISCUSSION

The major findings of this study are as follows: (1) the percentage of adverse events after mitral repair for DMR was 6.6% (20/301 patients) and the overall 5‐year freedom from adverse events rate was 84.0%, (2) conventional mitral hemodynamic parameters were not associated with adverse events, (3) patients’ age, postoperative LVEF and mean TMPG/LVSV were identified as independent risk factors for adverse events, (4) risk stratification by mean TMPG and LVEF reflected the midterm outcomes, predictively, and (5) although a full ring and smaller size of annuloplasty were associated with elevation of flow adjusted TMPG, the type of annuloplasty prosthesis (band or ring) did not have an influence in patients with a larger size (≥32 mm).

In previous studies, FMS, defined as elevation of TMPG, was associated with quality of life, atrial fibrillation, and left atrial remodeling after repair of DMR. Several studies reported that smaller prostheses may result in a higher mean TMPG, and may inhibit reverse remodeling of the left atrium, which caused a risk of late atrial fibrillation.^8^, ^19^, ^20^ Stress echocardiogram showed that a slight elevation of TMPG can be a risk for postoperative impaired exercise capacity and quality of life.^6^, ^8^, ^21^ To improve operative quality of mitral repair for DMR, elevation of TMPG must be avoided; however, the cut‐off value of mean TMPG ranged from 3.0 to 5.0 mmHg, and the definition of FMS, which can be a cause of cardiac adverse events, is still undetermined. Additionally, TMPG is strongly influenced by transmitral flow and the clinical impacts of elevated TMPG are still controversial.^22^ In the present cohort, mean TMPG ranged from 0.74 to 9.4 mmHg and had a significant positive correlation with stroke volume (*r* = .25, *p* < .001). Although isolated mean and peak TMPG was not a predictor of cardiac adverse events, TMPG adjusted by flow (LVSV or LVEF) was identified as a significant predictor and may have a potential to be a more sensitive indicator of FMS associated with clinical outcomes. The cut‐off value of mean TMPG/LVSV was 0.041, therefore, mean TMPG should be controlled to less than LVSV × 0.041 to avoid flow adjusted FMS. As shown in Figure 3, the normal LVEF, low gradient group had a good prognosis throughout the follow‐up period, whereas, in the low LVEF group, postoperative increased TMPG can prognosticate devastating clinical courses. The number in the low LVEF, high gradient group was small, but the outcome of this group was significantly poor. Of note, although the short‐term (0–2 years) outcomes of the normal LVEF, high gradient group appears to be good, its midterm (3–6 years) outcomes are almost equal to that of low LVEF groups.

Regarding the risks of FMS, smaller‐sized annuloplasty prostheses and complete rings reduce valve area and anterior leaflet motion, which may result in FMS.^6^, ^8^, ^21^, ^23^ These findings were well congruent with the results of the present study. Accordingly, in patients with larger annuloplasty prostheses (≥32 mm), the mitral hemodynamic parameters were equivalent between bands and rings. A full ring with a size ≥32 mm may be recommended to avoid FMS after mitral repair for ethnic Japanese patients with a small BSA in this cohort. In our present study, there was no significant correlation between repair techniques and flow adjusted TMPG. It is difficult to assess direct quantitative comparison of the surgical reconstruction techniques with the measurement of transvalvular pressure gradients. Several studies, which compare the use of neochordoplasty and leaflet resection for posterior leaflet prolapse or flail, reported the advantage of neochordoplasty in the preservation of the function of the native leaflet.^24^, ^25^ Jahren et al.^26^ examined the effects of different surgical methods on mitral valve hemodynamics in an experimental ex vivo porcine model. They concluded that neochordoplasty with or without ring annuloplasty was the only reconstruction technique to achieve native physiological hemodynamics.^26^ Further precise evaluation of the influence of techniques on the flow adjusted mitral hemodynamics is required. In summary, the standardized definition and clinical impacts of FMS are still undetermined, therefore, concepts of adjusted transmitral flow potentially could redefine FMS after repair of DMR.

### 4.1 Study limitations

There were several limitations. First, this study was a retrospective observational study in a single center. Therefore, there was a selection bias to decide the techniques of repair and products for mitral annuloplasty. Additionally, the influence of atrial fibrillation may not have been eliminated. Second, this cohort included physically small Asian patients, therefore, appropriate annuloplasty size selection may be different with a variant population. Finally, stress echocardiogram may be a better option for detailed evaluation. However, a stress echocardiogram cannot be performed routinely for all patients in all institutes. Therefore, we used resting echocardiographic parameters for simple and reproducible data.

## 5 CONCLUSIONS

TMPG is commonly emphasized to evaluate FMS. However, the definition and clinical impacts of FMS are still unclear. Adjustment by flow has a potential to advance a pressure gradient to a more sensitive indicator of FMS associated with clinical outcomes after repair of DMR. Although smaller complete rings may be useful to control regurgitation, they can cause a risk for FMS.

## CONFLICT OF INTERESTS

The authors declare no conflict of interest.

## AUTHOR CONTRIBUTIONS

*Conception or design of the work*: Arudo Hiraoka and Akihiro Hayashida. *Data collection*: Arudo Hiraoka, Toshinori Totsugawa, and Misako Toki. *Data analysis and interpretation*: Arudo Hiraoka. *Drafting the article*: Arudo Hiraoka and Genta Chikazawa. *Critical revision of the article*: Akihiro Hayashida, Hidenori Yoshitaka, and Taichi Sakaguchi. *Final approval of the version to be published*: Arudo Hiraoka.
